# Acupuncture induced pneumothorax: A case report

**DOI:** 10.1002/ccr3.2789

**Published:** 2020-03-18

**Authors:** Ninadini Shrestha, Bipin Karki, Pramesh Sunder Shrestha, Rupesh Gami, Subhash Prasad Acharya, Subodh Sagar Dhakal

**Affiliations:** ^1^ Department of Anaesthesiology Maharajgunj Medical Campus Institute of Medicine Kathmandu Nepal; ^2^ Department of Critical Care Medicine Om Hospital and Research Center Kathmandu Nepal

**Keywords:** acupuncture, complication, pigtail drainage, pneumothorax, traditional medicine

## Abstract

Various forms of alternative medicinal practices are gaining popularity. With this, there will be rise in the complications arising from these practices. Acupuncture is also such practice which though safe can rarely cause life‐threatening complications.

## Graphical abstract

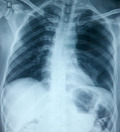

## 1 INTRODUCTION

Acupuncture is a form of traditional medicine that is regaining it is popularity as an alternate medicine. Although it is very safe, rare life‐threatening complications can occur after it. This is a case report of a life‐threatening pneumothorax resultant from an acupuncture therapy.

Acupuncture is a traditional form of medicine which has been regaining popularity as an alternative medicine over the decades, worldwide. Though it originated from China, it is now practiced worldwide, even in countries where the modern evidence‐based medicine is the main form of medicine practiced.^1^ It is used for varieties of illnesses, but is mainly popular for pain therapy. Increasing popularity and practice of acupuncture have increased the safety concerns associated with it. Serious and life‐threatening acupuncture related complications, though rare, have been reported.^2^ We report a case of a serious adverse event of pneumothorax following acupuncture therapy.

## 2 CASE REPORT

**Figure 1:**
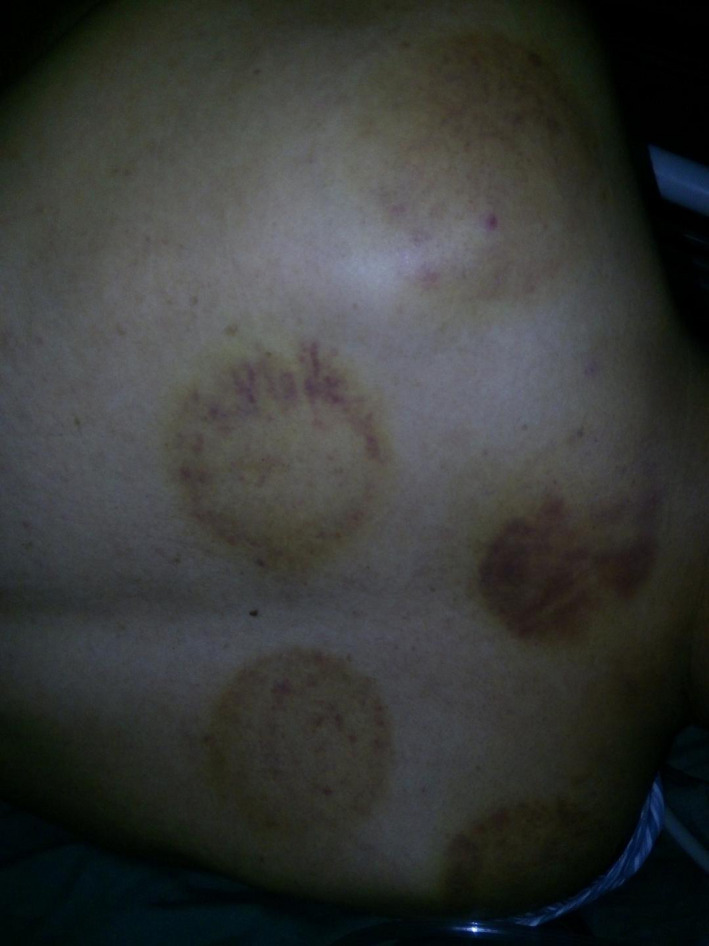
The marks from the acupuncture and cupping therapy from the day before

**Figure 2:**
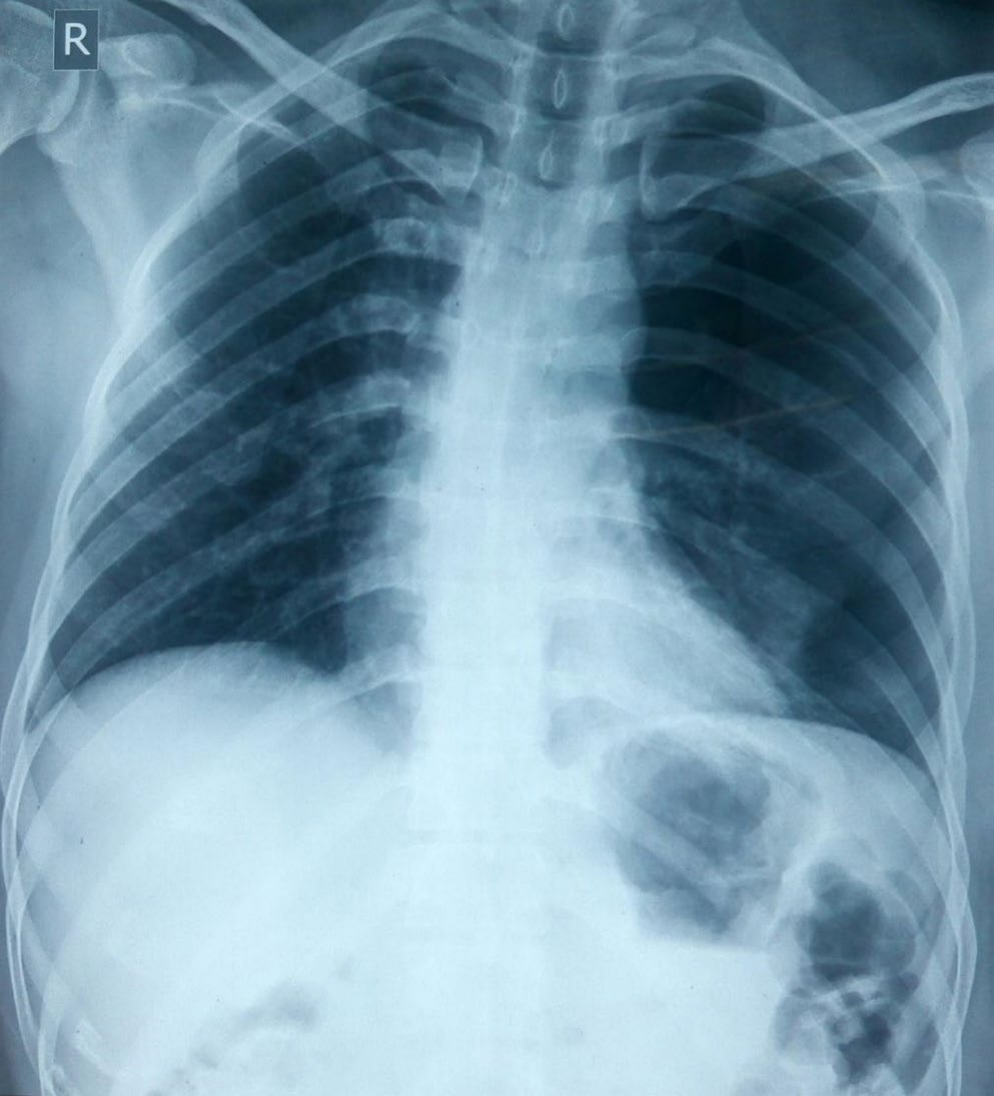
Chest X‐ray showing pneumothorax on the left side with no mediastinal shift

A 30 years male presented to the emergency department with shortness of breath for one day. It was gradual in onset but had increased to such severity that he had to seek hospital care. There was no significant past history of illnesses except for chronic back pain for which he had been under regular acupuncture therapy which consisted of needling and cupping therapies. His last session was just one day prior on an out patient's basis following which he had gone home. However, it had already been more than 20 hours postacupuncture. On examination, patient's heart rate was 100 beats per minute, blood pressure was 140/90 mm of Hg, respiratory rate was 40 breaths per minute and an arterial hemoglobin saturation of 88%, in ambient air. Oxygen therapy with a face mask was instituted. There was hyper‐resonance on left side of chest on percussion and severely diminished air entry on that side. On inspection of his back, he had superficial discoid bruises resulting from his last session the day prior (Figure 1) A chest X‐ ray was obtained (Figure 2).

**Figure 3:**
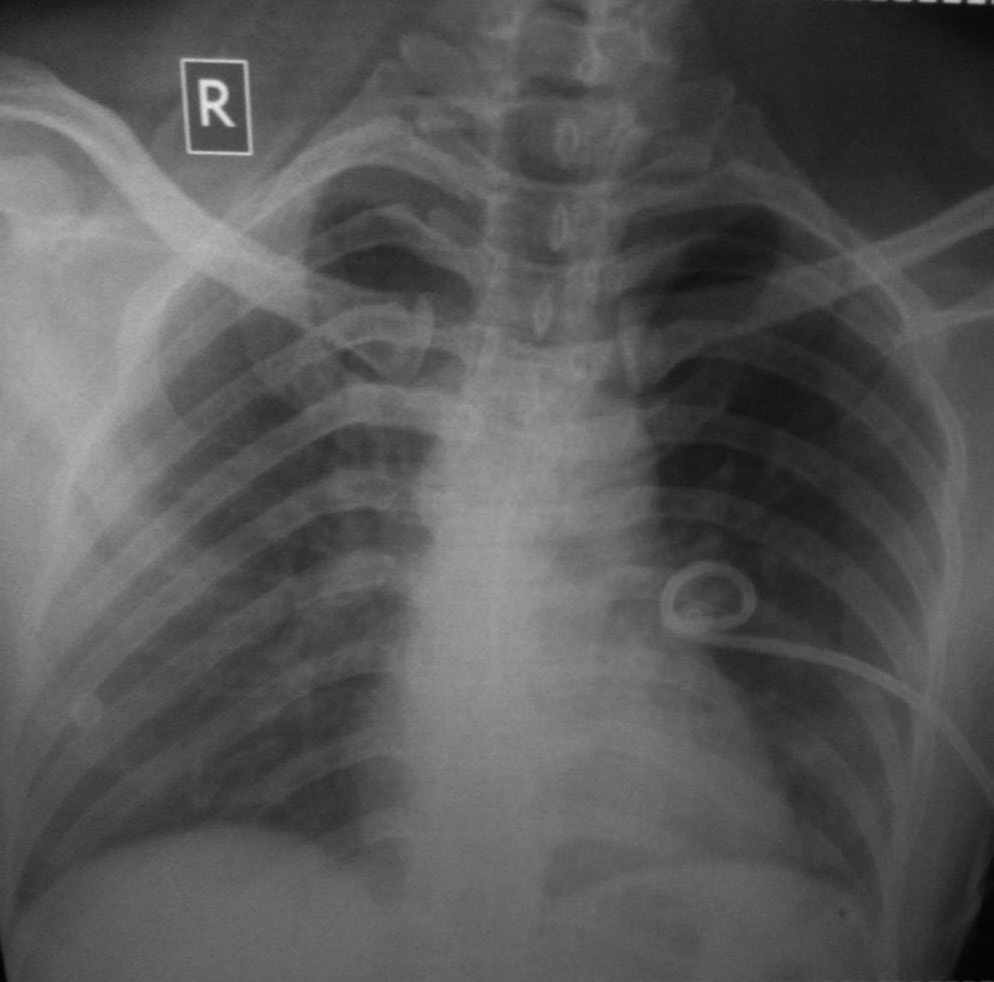
Chest X‐ray showing pigtail in situ with a fully expanded left lung

A diagnosis of left‐sided pneumothorax was made. A 14Fr pigtail catheter was inserted in the fifth intercostal space under local anesthesia in the mid‐axillary line and connected to a water seal drain. Movement of water column and bubbling was noted. The bubbling had stopped by the next morning. An X‐ray chest was obtained which showed a fully expanded left lung (Figure 3). The chest tube was removed on the third day, and patient was discharged on day four.

## 3 DISCUSSION

Adverse effects resulting from acupuncture are mostly very mild. Most of the side effects which have been reported have been found to be from the psychological aspects of the patients like dizziness and syncope. However, major side effects like pneumothorax, cardiac tamponade, infections, and even death have been reported. With the increase in the number of acupuncture patients and practices, the complications of these practices can be expected to rise. The incidence of serious adverse events has been found to be very low, 0.05 per 10 000 treatments, of which pneumothorax has been reported to be the most common adverse event even accounting for deaths.^2^, ^3^

The sequence of events in our patient points to the direct temporal relation between acupuncture and pneumothorax. While most of iatrogenic causes of pneumothorax are sudden in onset, our patient's symptoms had started quite some time later. This could be because of the size of the bronchopleural fistula created by the acupuncture needle. A small pneumothorax can be completely asymptomatic or become symptomatic upto days after the event. Similarly, a defect that has a ball valve mechanism can lead to rapid deterioration in the clinical status of the patient leading to immediate threat to life.^4^

A pigtail was inserted which lead to a full expansion of the lung and the patient was successfully discharged. Both chest tubes and pigtails can be used to treat pneumothorax. However, pigtails are easy to insert, have lower complication rates, are associated with earlier successful removal and lower length of hospital stay.^4^, ^5^

## 4 CONCLUSION

With an increase in the alternative forms of medicine practices, complications are also bound to increase. Pneumothorax can be a life‐threatening complication following acupuncture which should always be suspected in any patient who becomes short of breath following therapy.

## CONFLICT OF INTEREST

None.

## AUTHOR CONTRIBUTIONS

PSS and RG: involved in the patient care. They prepared the initial draft of the case report. NS and BK: responsible for the literature review and manuscript proof reading. SSD and SPA: responsible for the review and supervision of the entire work. All the authors reviewed the final draft of the case report.
